# Rice Husk with PLA: 3D Filament Making and Additive Manufacturing of Samples for Potential Structural Applications

**DOI:** 10.3390/polym16020245

**Published:** 2024-01-15

**Authors:** Gabriela Barreto, Santiago Restrepo, Carlos Mauricio Vieira, Sergio Neves Monteiro, Henry A. Colorado

**Affiliations:** 1 CCComposites Laboratory, Engineering School, Universidad de Antioquia (UdeA), Calle 70 No. 52-21, Medellin 050010, Colombia; gabibarreto93@gmail.com (G.B.); santiago.restrepol@udea.edu.co (S.R.); 2 Advanced Materials Laboratory, LAMAV, UENF—State University of Northern Rio de Janeiro, Av. Alberto Lamego, 2000, Campos dos Goytacazes 28013-602, RJ, Brazil; vieira@uenf.br; 3 Military Institute of Engineering, IME, Praça General Tibúrcio 80, Urca, Rio de Janeiro 22290-270, RJ, Brazil; snevesmonteiro@gmail.com

**Keywords:** additive manufacturing, polymer composites, PLA, 3D printing, filaments, FDM, polymer composites, rice husk

## Abstract

Additive manufacturing has garnered significant attention as a versatile method for fabricating green and complex composite materials. This study delves into the fabrication of polymer composites by employing polylactic acid (PLA) in conjunction with rice husk as a reinforcing filler. The filaments were made by an extruded filament maker and then were used to make tensile and impact samples by another extrusion technology, fused deposition modeling (FDM). The structural and morphological characteristics of the composite materials were analyzed using scanning electron microscopy SEM. Results show that both the filament and samples are very reliable in producing polymer parts with this rice husk solid waste. This research contributes to increasing materials’ circularity and potentially creating a local social economy around rice production, where this waste is not much used.

## 1. Introduction

Additive manufacturing (AM), also known as 3D printing (3DP), is the process of creating parts layer by layer [1]. AM technologies are categorized by ASTM F2792 [2] into seven distinct groups: material extrusion (ME), binder jetting (BJ), material jetting (MJ), sheet lamination (SL), vat photopolymerization (VP), powder bed fusion (PBF), and directed energy deposition (DED). This technology has evolved significantly from its initial role in rapid prototyping to become a pivotal method for producing functional end-use parts [3], offering distinct advantages, including the capability to fabricate intricate, integrated designs swiftly and cost-effectively without the need for specialized tooling. It has found applications across diverse industries, such as the automotive [4], biomedical [5], aerospace [6], building materials [7], education [8], armor and military [9], electronics [10], dental [11], and public health [12] industries, among many others.

Among the spectrum of AM techniques, fused deposition modeling (FDM) stands out as the most widely adopted method due to its reliability, low cost, and simplicity [13].

FDM involves melting a thermoplastic polymer, which is then extruded through a nozzle and deposited layer by layer onto a build platform, a process that has garnered significant success in the industry, thanks to its robustness and cost-efficient design [14]. The end products made from pure polymers, though, frequently lack the necessary strength to create fully functional engineering components, limiting the broader utilization of this technique. To tackle this challenge, strengthening elements like fibers are incorporated into the polymer matrix during the printing process, yielding a composite structure that typically demonstrates enhanced mechanical properties [15].

Thermoplastic, when integrated with additives (such as carbon fibers), can be conveniently procured from the market. Alternatively, individuals can produce personalized filaments utilizing composite materials with accessible and small-scale filament extrusion equipment. Acrylonitrile butadiene styrene (ABS) and polylactic acid (PLA) and acrylonitrile butadiene styrene (ABS) stand as frequently employed thermoplastic resources to the FDM process, largely due to their low melting points [16].

Derived from sustainable sources at a cost-effective rate, the thermoplastic polyester named polylactic acid (PLA) exhibits unique advantages. Boasting a low melting point, PLA readily accommodates various FFF machinery. Its extrusion temperature surpasses that of prevalent polymeric counterparts like ABS (acrylonitrile butadiene styrene), PEEK (polyether ether ketone), and PETG (polyethylene terephthalateglycol), while often delivering superior tensile strength and elastic modulus. Moreover, PLA shines as a biodegradable option with a minimal carbon footprint and negligible smoke emissions during extrusion. Its medical utility is underscored by its non-metabolically harmful nature [17].

Polylactic acid (PLA) has garnered significant attention as an aliphatic thermoplastic polymer derived from renewable sources like sugarcane and corn. PLA’s versatile properties, spanning biodegradability, broad availability, eco-friendliness, antibacterial attributes, and commendable mechanical and thermal qualities, position it as a compelling candidate for diverse sustainable applications [18].

In comparison with traditional petroleum-based polymers like polystyrene (PS), PLA exhibits noteworthy mechanical strength and toughness, albeit with challenges like impact resistance and thermal limitations [19]. Ongoing research seeks to optimize these characteristics for expanded applications, particularly in agriculture [20], packaging [21], and medical realms [22].

Within the global push for sustainable alternatives and the depletion of petroleum reserves, PLA emerges as a fitting choice, driving exploration in biocomposites for various applications, including biomedical and food packaging. Despite inherent limitations, PLA’s intrinsic properties align with specific needs, emphasizing its potential in niche sectors. In essence, PLA signifies a significant stride in the realm of sustainable thermoplastics, embodying a balance between eco-friendliness and versatile functionality across diverse industries [18].

This biopolymer is sourced from renewable origins like corn, potatoes, sugarcane, cassava roots, and sugar beets. It is important to note that the carbon dioxide generated during PLA’s disposal, incineration, or biodegradation does not contribute to the overall atmospheric carbon dioxide levels. Consequently, the environmental impact during PLA’s production and disposal, including its carbon footprint, is considerably lower when contrasted with petrochemical-based polymers [23].

Integrating particles, fibers, or nanomaterial enhancements into polymers allows for the creation of composite materials characterized by exceptional performance and functionality [24]. These composites, strengthened by short and continuous fibers [25], offer an appealing combination of ease of manufacturing, cost-efficiency, and superior mechanical characteristics. In the context of FDM processing, filaments are produced through a two-step procedure, initially involving the blending of polymer pellets and fibers, followed by extrusion to create the filament [26].

There is an increasing number of research incorporating solid waste materials into materials such as composites [27] or construction materials [28] to reduce the pollution associated with some processes and to have an economic benefit as well. Additive manufacturing has not been the exception in developing strategies towards a more sustainable industry [29], and now, printed cement with organic wastes [30], ceramics with hazardous waste [31], polymer matrix filaments incorporating cashew nutshell wastes [26], and sawdust have been developed for additive manufacturing [32].

Numerous studies have explored the use of short fibers, like chopped carbon, basalt, or glass fibers as reinforcements, resulting in the enhancement of mechanical properties [33,34,35]. So far, none have studied PLA with rice husk, which is a prevalent byproduct of rice cultivation, and contributes significantly to agricultural waste. 

Globally, annual rice production yields over 750 million tons of grain, accompanied by approximately 150 million tons of husk [36], which due to the production amount, is not fully properly used. This substantial waste generation requires multiple strategies to increase the material’s circularity, and additive manufacturing of these composites [37] is one feasible alternative to increase the use of rice husk.

Rice husk contains approximately 50%, 25–30% and 15–20% of cellulose, lignin, and silica contents, respectively [38]. In a recent investigation conducted by Singh et al. (2023) [39], the application of rice husk as a substitute for the sand component in cement concretes yielded a significant improvement in their mechanical performance. Similarly, Shirgire et al. (2023) [40] demonstrated that concretes incorporating rice husk displayed superior performance when compared to their conventional counterparts. Moreover, Mohamed et al. (2020) [41] successfully formulated a polymer composite using rice husk fibers embedded in a polypropylene matrix.

Building upon these findings, the current research extends its focus to explore the utilization of rice husk ash as a reinforcing agent for PLA. The objective is not only to contribute to the broader utilization of rice husk as a byproduct, thereby reducing its environmental impact, but also to investigate the potential effects it may have as a reinforcement for PLA. 

This research explores the feasibility of producing composites of PLA with rice husk as a reinforcement for AM, including several characterization techniques for the understanding of the filaments and samples’ performance, but also for establishing the structure–property relation of these materials.

## 2. Materials and Experimental Methods

The materials, polylactic acid (PLA) in pellets from Estra SA from Medellin, Colombia, and rice husk from Prila SAS, from Medellin, Colombia, were used as raw materials in this research. The rice husk was first ground in a jaw mill to reduce the particle size, then dried, and sieved through a 200-mesh sieve.

**Figure 1:**
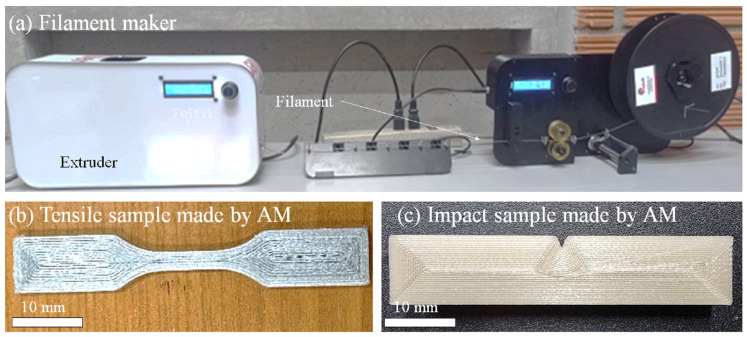
(**a**) Filament maker used to produce the PLA-rice husk filaments; (**b**) samples made by AM with a FDM printer; and (**c**) Izod Impact strength samples made by AM as well.

To manufacture PLA-rice husk filaments, pelletized PLA was manually blended for 5 min seeking homogeneity, and with varying proportions of rice husk powder at different weight percentages—0%, 0.5%, 1.0%, and 2.0%—four distinct compositions were yielded. The homogenized mixture was fed into the extruder machine, where heat-induced melting of the blend was carried out, facilitating an efficient fusion of PLA and rice husk. This approach was deemed more suitable for our experimental setup. The filament extrusion systems utilized, the Felfil Evo (Figure 1) attached to the Felfil Spooler, were both manufactured by Felfil in Turin, Italy.

About 50 m of filament was manufactured for each composition. It is important to mention that the rice husk was only well dried and not further treated because this research intends to motivate real solutions for chemicals or any other treatments that can increase the product costs and thus make the solution not feasible. The Felfil Evo was powered on, fed with the compositions to be extruded, and following the manufacturer’s instruction, was set to 205 °C and 0.0 RPM for 20 min before the extrusion process began. The Felfil Evo can operate continuously for a maximum of 4 h. After this period, it must be turned off for 2 h to rest. Upon restarting, the same procedure of setting the temperature to 205 °C with 0.0 RPM for 20 min is required to preserve the equipment. 

Each of the four compositions were extruded at three different temperatures: 177 °C, 187 °C, and 197 °C. The chosen temperatures align with the guidelines outlined in the Felfil Evo manual for PLA extrusion i.e., 187 °C, and the lower and higher temperatures were used to adjust the flow of material through the added fibers. The pull speed initially set at 0.8 m/m was adjusted along with RPM to stabilize the filament diameter. Speed adjustments should be made based on temperature and the amount of rice husk, with continuous testing required until a stable flow is achieved, aiming for the desired filament diameter set at 1.75 mm, which would be the optimum diameter for the subsequent 3D printing process. Therefore, RPM and pull speed were fine-tuned with variations between 5 and 8 RPM and between 0.50 and 0.90 m/s of pull speed, depending on the specific filament. These slight adjustments were filament-dependent, while all other parameters remained constant. 

The Felfil Spooler was set to Manual Mode, with the desired filament diameter set at 1.75, ideal for many 3D printers. The fan speed was set to 150 out of 255 maximum speed (8-bit format), which is around 58.8% of the maximum speed given by the machine. After the extrusion process, the filaments were vacuum sealed using a vacuum apparatus. These sealed filaments were then stored in plastic bags for subsequent testing. 

The morphological analysis of the rice husk and its printed composites was performed using scanning electron microscopy with energy dispersive X-ray spectroscopy (SEM-EDS) at the Universidad de Antioquia, Colombia. Samples were all gold sputtered before the observation. 

The filaments underwent tensile strength testing at 5 mm/min at room temperature, using a Shimadzu apparatus. Also, typical dog-bone-shaped specimens were fabricated using an Anet A8 FDM 3D printer and their tensile strength was determined following the ASTM D638 standard [42]. 

Samples for the Izod Impact tests were also made by additive manufacturing, see Figure 1, and were tested on a CEAST apparatus following the ASTM D256 standard [43]. For the tensile strength tests, 5 specimens of each composition were 3D printed with a thickness of 2 mm, while 3 specimens of each composition were printed for the Izod Impact test. The fractured surfaces that underwent the tensile test were further analyzed by SEM, while the fractured surfaces that underwent impact tests were 3D scanned. 

The printing process parameters included a layer thickness of 0.2 mm, a printing speed of 60 mm/s, a nozzle temperature of 200 °C, and a bed temperature of 60 °C. The specimen designs were created using 3D software Inventor Professional 2023, and they were subsequently laminated with the software Cura Slicer, version UltiMaker-Cura-5.6.0. Then, the files were uploaded to the 3D printing machine with a Software named PronterFace version Primtrun 2.0.1, and the part was produced. The fractured surfaces of the impact fractured samples were further analyzed using a 3D scan type GOM 1 from ZEISS (Oberkochen, Germany).

## 3. Results and Discussion

**Figure 2:**
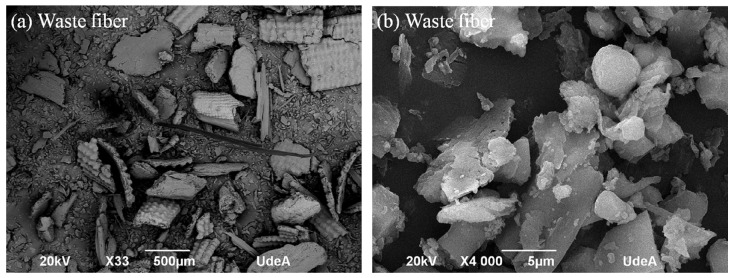
Raw rice husk powder at (**a**) 33×, (**b**) 4000×.

**Figure 3:**
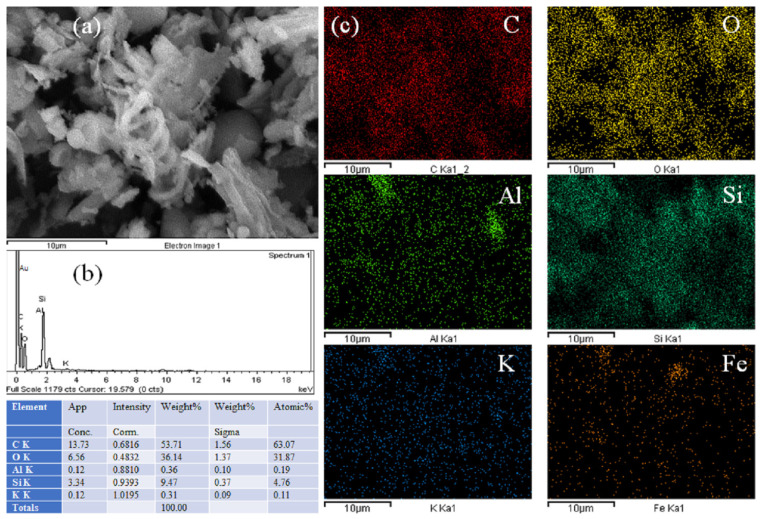
SEM results for the raw waste powder rice husk showing (**a**) the scanned area; (**b**) elemental composition and spectrum; and (**c**) all composition maps for elements C, O, Al, Si, K, and Fe.

**Figure 4:**
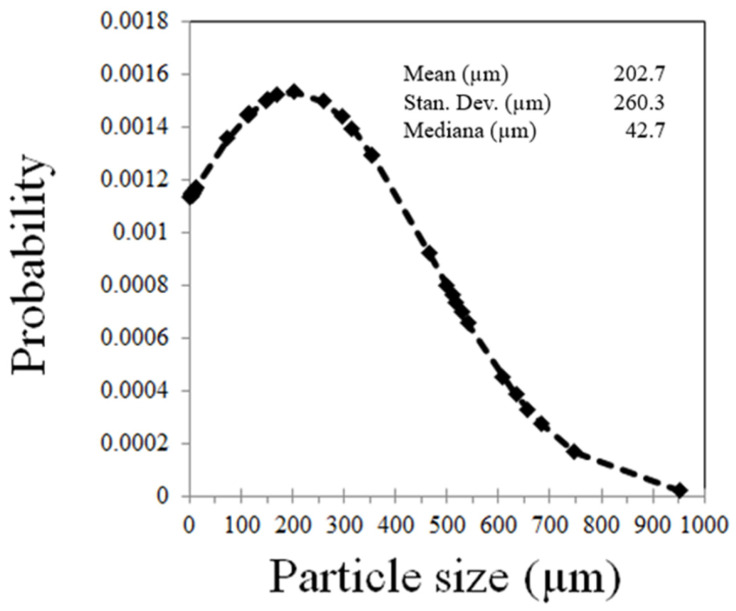
Particle distribution of the rice husk powder.

Figure 2 shows images of the rice husk powder at two different magnifications, 33 and 4000×. The main characteristic is that these particles are quite variable in shape, from needle to rounded, and with sizes from about 1 to 50 µm. Moreover, Figure 3 shows a spiral structure, common in natural fibers. This image shows compositional maps, where silicon oxide (Si map), and aluminum oxide (Al map) reflect possible quartz and other minerals in the composition. Figure 4 shows a non-normal distribution, which is basically due to the hammer mill effect and thereafter, the sieved process through a 200-mesh sieve gave a combination of large and small particles, observable both in the SEM images of Figure 2, with a mean of 202.7 µm and a standard deviation as high as 260.3 µm. Also, silica particles were identified to be part of the rice husk ash composition, which are naturally solid, exhibiting amorphous configurations akin to cristobalite and containing minimal traces of crystalline quartz [44]. The transformation of crystalline rice husk into amorphous forms occurs exclusively through high temperatures. Therefore, the disparity in particle size is attributed to the elevated combustion temperature of rice husk [38].

**Figure 5:**
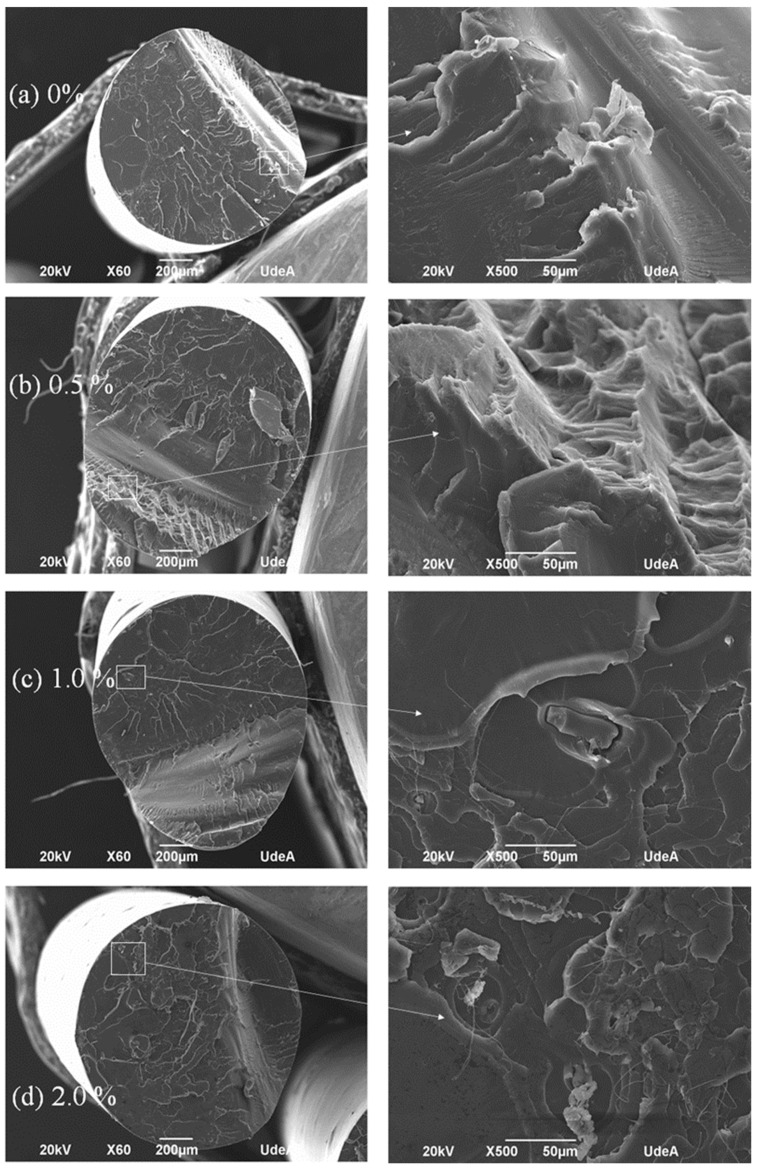
SEM images for the filaments fabricated with the extruder made of PLA and (**a**) 0.0 wt%, (**b**) 0.5 wt%, (**c**) 1.0 wt%, and (**d**) 2.0 wt% of rice husk powders.

Upon the filament fabrication, see Figure 1, the filaments were observed in the SEM. Figure 5 shows a cross-section view of the filaments, after a fragile fracture using liquid nitrogen. Besides the fracture temperature, all of the samples revealed a ductile fracture, which is due to the great elongation at the break of PLA; see Figure 5a showing PLA with no rice husk (0%). After adding 0.5% of rice husk, Figure 5b, the ductile behavior was maintained, and it was difficult to identify any fibers. When adding 1.0 and 2.0% of rice husk, it was possible to find some fibers clearly seen in the image insights, which show particles with limited adhesion to the PLA matrix. In general, it is observed that the fibers are well distributed because the few fibers that were added did not appear even in pairs; they were solely surrounded by the PLA matrix, although the adhesion between the fiber and the matrix did not always seem good, as observed in the magnification of Figure 5c.

Regarding details of the fractured surfaces, PLA is a quite ductile polymer, and besides these fractured surfaces that were made under liquid nitrogen, the typical fractured zones can be identified [45,46]: an initial crazing-shearing zone, a cutting zone, a brittle zone, and a tensile zone, at the end of the fracture. Probably because of the preparation, almost no dust is observed. Figure 5a for just PLA for instance shows flat crazing-shearing and cutting zones, up to about ¾ of the diameter, and then appears an abrupt fragile zone with an important change in the inclination, and then finishes with a small tensile zone. The small insight of the image shows the limits of these last zones. Figure 5b for 0.5% of rice husk shows the same zones, but in this case, the brittle zone is very clear and clean. Also, the crazing-shearing zone reveals craze fibrils, a product of severe plastic deformation. The magnified image shows the tensile zone. Figure 5c for 1.0% of fibers is similar in general but shows a clear spherulite at the beginning of the fracture and near the ground fibers. Some small fragments are observed at the end of the crazing-sharing zone, and there is a big crack in the limits of the brittle zone. The magnification selected for this sample shows the crazing-sharing zone with short rice dusk fibers and significant craze fibrils, some with lengths over 100 µm. Last, Figure 5d for 2.0% of fibers shows similar zones to 1.0% of fibers, but the brittle and tensile zones seem more intense, which can be expected due to the fiber content increase. The magnification of this image shows details of a spherulite and large craze fibrils from the crazing-sharing zone, with some of them torn and thicker than the other samples.

**Figure 6:**
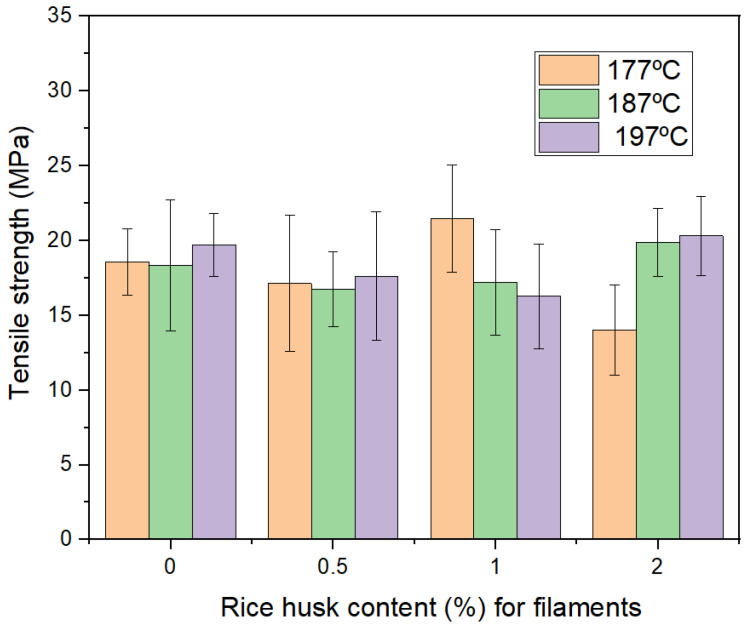
Summary of tensile strength for filaments.

The single filament tensile tests from Figure 6 show the tensile strength results for filaments made at different temperatures and with different rice husk contents added. The standard deviation shown by the error bars is significant for all the samples, which can be explained by the defects produced during the extrusion process, such as voids and possible particle inhomogeneities. Samples with 0% of waste have 18.3, 18,7, and 19.6 MPa average strength for filaments made at 177, 187, and 197 °C, which reveals an increasing trend with the extrusion temperature, which can be understood as better melting of PLA as the temperature is increased. 

Although all the fibers were used as ground fibers, this is without further treatment; for samples with 1 and 2 wt% of waste, the tensile strength value increases at the highest processing temperatures, 187 and 197 °C. This can be explained as better impregnation of resin in the fibers at higher temperatures when the thermoplastics have less viscosity, which corresponds with more chemical bonds from fiber to matrix, therefore improving the tensile strength. From the results, 2.0 wt% of rice husk seems to be the upper limit after the strength starts to decrease. For samples with 2.0 wt% of rice husk made at 177 °C, the tensile strength decreases, which can be explained as more defects (voids and lack of resin impregnation) due to more fibers in the mix. 

When 0.5% of fibers were added, the strength showed a significant decrease: 17.1, 17.3, and 18.4 MPa average strength for filaments made at 177, 187, and 197 °C. This strength drop with the fiber content is explained by the presence of rice husk particles, acting as stress concentrators, and in cases such as the lack of adhesion shown in Figure 5c, particles can be in some cases decreasing (as voids) the overall strength of the polymer matrix. With few exceptions, these trends of strength decrease as fiber contents increase and increase as extrusion temperature increases is maintained for filaments with 1 and 2% of rice husk, each processed at 177, 187 and 197 °C.

**Figure 7:**
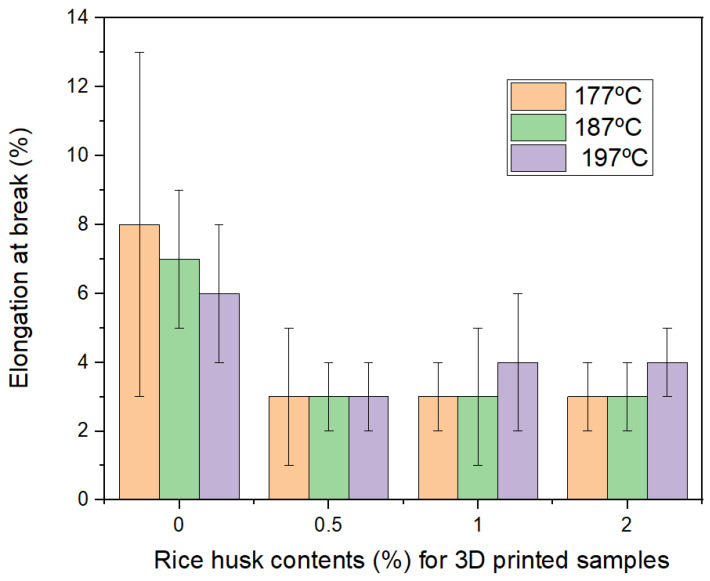
Summary of elongation at the break for filaments.

Figure 7 describes the elongation at the break with the rice husk content and with temperature as well. Clearly, these tiny fibers strongly decrease the elongation at the break, almost three times, when compared to the composites with the neat PLA matrix. There is also a slight increase in elongation at the break when the extrusion temperature is increased for samples with rice husk, which can be explained as better impregnation of the PLA matrix in rice husk at a higher temperature. 

**Figure 8:**
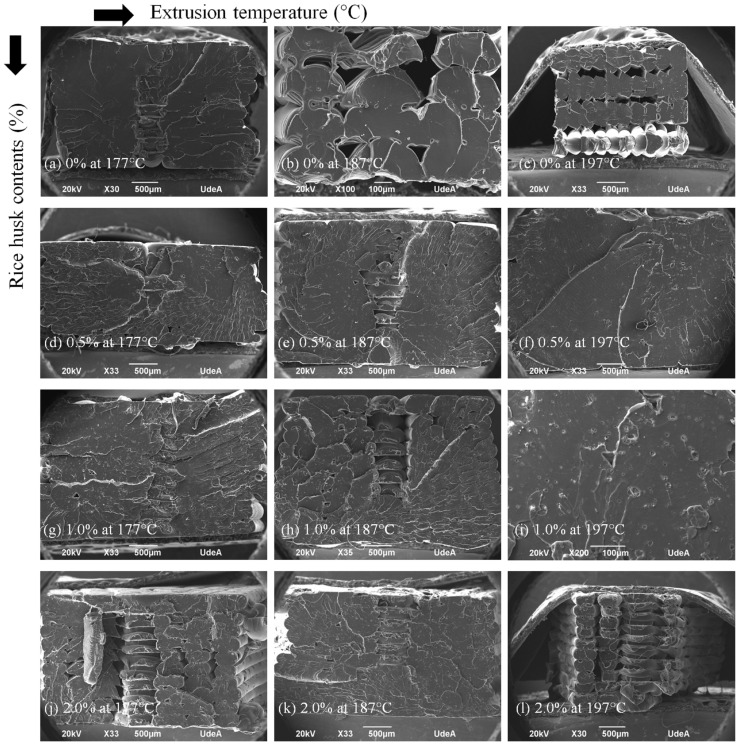
SEM cross-section of the tensile strength samples.

After the filaments were tested, typical dog-bone-shaped specimens were fabricated with FDM 3D printing apparatus. SEM cross-section view images of the samples are shown in Figure 8. Samples with 0% rice husk showed fewer defects when printed at 177 °C. Samples with 0.5 and 1.0 of fibers showed better samples overall, with fewer voids, which must be due to a fiber effect. Even samples with 2 wt% of fibers and few voids did not show this.

**Figure 9:**
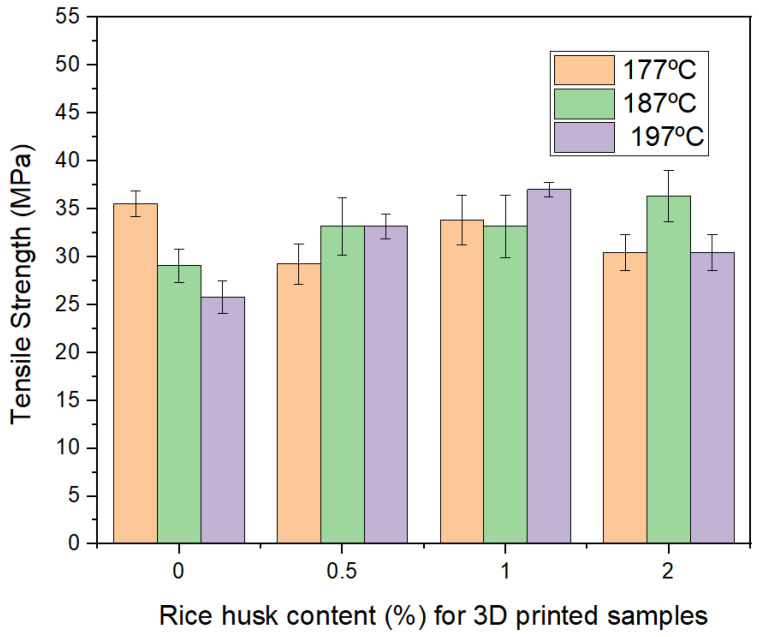
Summary of tensile strength of 3D printed samples.

The summary of the tensile strength for 3D printed samples with the filaments previously analyzed is presented in Figure 9. For samples without waste, 0% of rice husk, the strength is reduced as the processing temperature is increased, which is well understood when Figure 8a–c are observed, and as explained before, the increase of temperature produces more voids, probably due to over melting. For samples with rice husk (0.5, 1.0, 1.5, and 2.0), there is no clear trend, although overall it seems that the fiber content can increase strength. 

**Figure 10:**
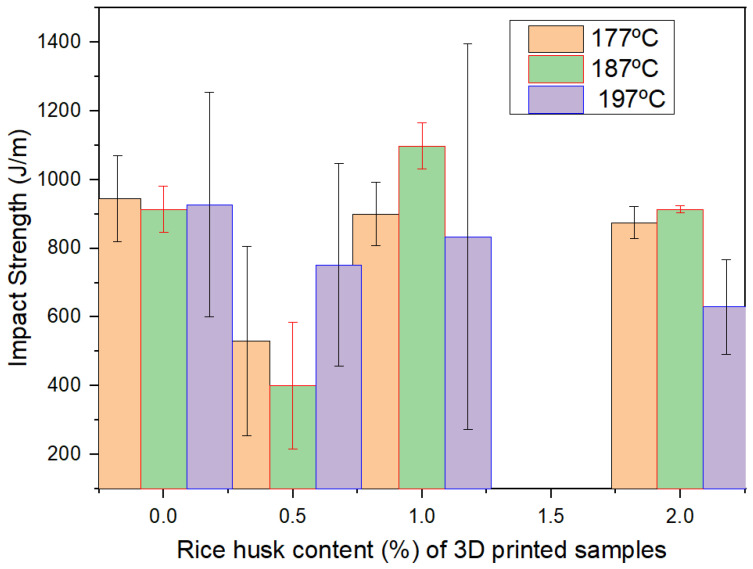
Summary of impact results.

In the case of impact, see Figure 10, the trend is also difficult to see, although the values are kept almost in the same range (mean values between 400 and 1200 J/m) besides the increase in the waste contents, and the defect variability.

**Figure 11:**
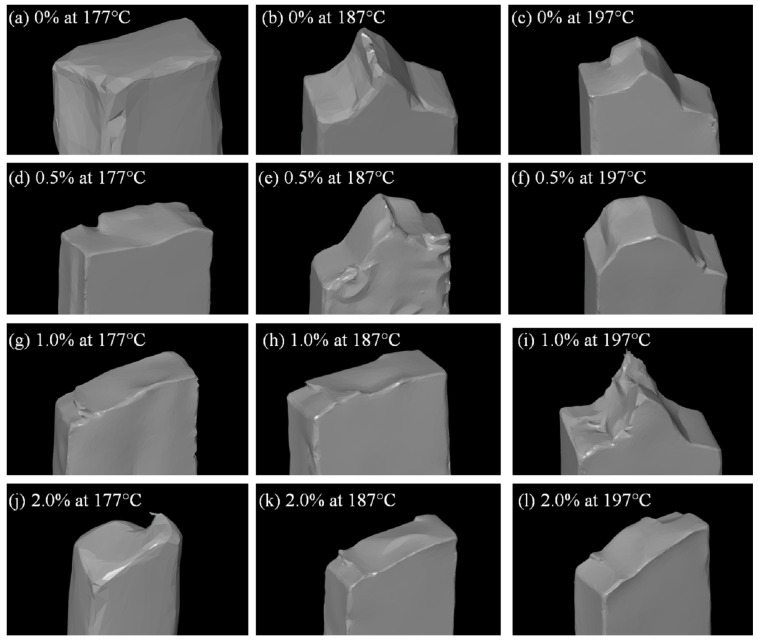
3D scanning images of some of the samples tested under impact.

Figure 11 shows typical fractured images after the tensile tests for the 3D-printed samples. In general, the lowest extrusion temperature showed a more fragile fracture, designated by the flat surfaces, see Figure 11a,d,g,j. As the temperature increased, in general, the fracture looked more ductile, showing very irregular fractured surfaces, such as Figure 11b,c,e,f,i. For samples with 2.0% rice husk, no clear ductile behavior is seen, which suggests that even these short fibers in contents as low as 2.0% in PLA are very significant for stresses and perhaps dynamic applications.

## 4. Discussion

This research has shown that rice husk can be fabricated for 3D printing filaments with percentages up to 2.0 wt% obtained in all mixtures over 25 MPa in tensile strength, and depending on the processed extrusion temperature, the mean strength can be improved with respect to the neat sample of extruded PLA, see Figure 6. The elongation at the break decreased more than 50% in all cases with respect to the PLA. The literature shows that additive manufacturing PLA by FDM has been reported in a wide range of tensile strength values, from about 15 to 72 MPa [47], which is quite variable and depends on the processing parameters, sample size, and other factors. As mentioned above, 25 MPa certainly is inside this range and an acceptable value. Elongation at the break has been reported with very similar values [48] for 3D printed samples of PLA with rice husk. These deformation values are expected to reduce with the addition of the powder because in tensile tests, the particles usually can reduce the values with respect to the pure polymer matrix, which has been reported in many particles reinforcing polymer matrix composites [49]. This same investigation [48] has shown PLA with rice husk with values over 30 MPa in tensile tests, which is explained as a more complex process of vacuum and particle preparation, which allows inclusion of very high particle contents. Upon these reported results, our investigation is quite good as the vacuum or other processing was not used, which clearly has sustainability advantages.

Also, nanocomposites of PLA with untreated rice husk at 4 and 6% [50] were made by an electrospun process, and although strength is reported to be doubled with respect to the neat sample, the results are not clearly presented. Other authors showed that combining PLA with rice husk 10–30% and clay 1–5% can increase tensile strength up to 33 MPa, although compression molding was used [51]. 

Finally, rice husk with HDPE and 65% waste obtained with a traditional extrusion process showed about 13.5 MPa [52] in tensile strength without chemical treatment, while it was 20.9 MPa when a polymer coupling agent was used. Considering that 3D printed samples can include more defects, the results presented in this investigation have very competitive values when compared to the traditional fabrication process, where voids can be more easily eliminated.

On the other hand, solid waste, and particularly organic waste, is a worldwide problem because it is directly related to population increase and consumerism and needs to be considered seriously for the future of humankind [53]. Although much of this waste is due to agriculture, a great amount of it is not properly used or recycled, which increases the problem of waste management in many countries with not enough landfills [54]. Another massive alternative for organic waste utilization is waste-to-energy [55], such as sawdust or pruning waste being used as fuel to provide heat in many processes. However, this solution seems to not be the best alternative due to some of the gases that can be generated [56] which is a concern aligned with the campaigns to decrease the CO_2_ footprint of manufacturing processes, and therefore cannot be the only solution for organic wastes. Thus, it is important to find alternative solutions, such as new materials incorporating solid wastes into parts traditionally made of other materials, such as waste materials in construction [57], and although for now, 3D printing is not as massive as other sectors in terms of materials amounts, if combined with wastes in construction and other structural materials, it could be a contributor for reducing pollution [58]. Implementing natural fibers in AM can not only be useful for the 3D printing sector, but also can be a CO_2_ footprint reducer, and moreover, a social economy for communities with farmers that produce these types of valuable wastes. This flexible economy is possible because of AM characteristics [59]. 

There are several aspects that are important to discuss in this research. Starting from the filament fabrication, this research found that depending on the temperature, materials combination, and extrusion speed, the filament fabrication must be changed to maintain the diameter. Moreover, the fiber contents completely changed the process parameters because of the thermal properties of the extruded materials, which validates the method of fabricating the samples at different temperatures. It is also significant that these results were obtained using waste fiber without chemical treatment, that is, only using thermomechanical processes during the entire process: grinding rice husk, drying, and extrusion. This makes not only the process simpler to implement elsewhere, but also more economically and ecologically sustainable than other processes reported before [60,61,62,63], which is key for large-scale and real implementation of processes [64]; this is particularly important in developing economies, where there is not much interest in technology investment but positively, there is cheap labor, which makes these processes a life solution for locals and contributes to the formal recycling sector [65]. Further improvement is feasible with smaller powder sizes and fiber functionalization processes aiming at rice husk—PLA interface strengthening, particularly for tensile and flexural applications. Large contents of waste in the matrix probably require vacuum optimization to avoid powder agglomeration and massive voids, which significantly decrease the composite properties. 

## 5. Conclusions

This research has shown the feasibility of rice husk as a filler for PLA filaments for fused deposition modeling (FDM). For filaments, it was found that as fiber content increases, the filament strength was kept almost at the same values. Also, it was found that with the increase in the extrusion temperature, the tensile strength increased. Moreover, for the printed samples, particularly when the rice waste was added, the porosity did not change significantly. The impact strength was also kept well for all fiber contents. 

This solution was kept as a green alternative as much as possible, since the waste organic material used as reinforcement of PLA avoided chemical processing, the functionalizing of the particle surface or any other treatment was reduced to a minimum. This is not only sustainable for the environment but it is also competitive economically since chemical treatments can significantly increase processing costs. 

Further improvements can be made if the particle contents can be increased via processing optimization without a detrimental effect on the tensile properties, for which fiber functionalization for better fiber to matrix adhesion could be explored.

The journey of developing PLA filaments infused with rice fibers via extrusion not only marks a significant achievement but also opens exciting avenues for future exploration. Looking ahead, the potential applications of our eco-friendly PLA filament extend into sectors like construction, packaging, and 3D printing, presenting enticing opportunities in a growing market for sustainable materials. Beyond this, future investigations may delve into expanding the range of natural fibers, and creating filaments with distinctive properties. The nuanced understanding of fiber–matrix interactions promises tailored material characteristics, catering to a spectrum of industrial needs. In essence, the prospects ahead showcase the enduring impact of our study in steering materials engineering towards innovative, sustainable, and high-performance solutions.

## Data Availability

Data will be available upon request to the contact author.
